# Modeling the Temperature Dependence of Dynamic Mechanical Properties and Visco-Elastic Behavior of Thermoplastic Polyurethane Using Artificial Neural Network

**DOI:** 10.3390/polym9100519

**Published:** 2017-10-18

**Authors:** Ivan Kopal, Marta Harničárová, Jan Valíček, Milena Kušnerová

**Affiliations:** 1 Institute of Physics, Faculty of Mining and Geology, Vysoká škola báňská—Technical University of Ostrava, 17. Listopadu 15, 708 33 Ostrava, Czech Republic; marta.harnicarova@vsb.cz (M.H.); jan.valicek@vsb.cz (J.V.); milena.kusnerova@vsb.cz (M.K.); 2 Department of Numerical Methods and Computing Modelling, Faculty of Industrial Technologies in Púchov, Alexander Dubček University of Trenčín, Ivana Krasku 491/30, 020 01 Puchov, Slovakia; 3 Regional Materials Science and Technology Centre, Vysoká škola báňská—Technical University of Ostrava, 17. Listopadu 15, 708 33 Ostrava, Czech Republic; 4 Technical Faculty, Slovak University of Agriculture in Nitra, Tr. A. Hlinku 2, 949 76 Nitra, Slovakia

**Keywords:** thermoplastic polyurethanes, visco-elastic properties, dynamic mechanical analysis, stiffness-temperature model, artificial neural networks

## Abstract

This paper presents one of the soft computing methods, specifically the artificial neural network technique, that has been used to model the temperature dependence of dynamic mechanical properties and visco-elastic behavior of widely exploited thermoplastic polyurethane over the wide range of temperatures. It is very complex and commonly a highly non-linear problem with no easy analytical methods to predict them directly and accurately in practice. Variations of the storage modulus, loss modulus, and the damping factor with temperature were obtained from the dynamic mechanical analysis tests across transition temperatures at constant single frequency of dynamic mechanical loading. Based on dynamic mechanical analysis experiments, temperature dependent values of both dynamic moduli and damping factor were calculated by three models of well-trained multi-layer feed-forward back-propagation artificial neural network. The excellent agreement between the modeled and experimental data has been found over the entire investigated temperature interval, including all of the observed relaxation transitions. The multi-layer feed-forward back-propagation artificial neural network has been confirmed to be a very effective artificial intelligence tool for the modeling of dynamic mechanical properties and for the prediction of visco-elastic behavior of tested thermoplastic polyurethane in the whole temperature range of its service life.

## 1. Introduction

Thermoplastic polyurethanes (TPUs) belong to the family of thermoplastic elastomers, which combine mechanical properties of rubber based materials with the good processability and recyclability of thermoplastics. They represent linear segmented semi-crystalline multi-block copolymers, composed of an alternation of short stiff hard crystalline segments and rather long soft amorphous flexible chains. The hard segments are made from di-isocyanate, while the soft segments consist of long and flexible polyether or polyester chains, which mutually connect two hard segments. In particular, the hard segments act as multi-functional tie points working as both physical cross-links and reinforcing fillers. The domain structure formed by micro-phase separation of TPUs, due to the thermodynamic immiscibility or incompatibility between the hard and soft phase, endows these polymeric materials with their elastomeric properties, such that can be seen in cross-linked rubber networks [1,2]. The unique chemical structure of the TPUs results in a combination of interesting properties. They are characterized by a wide service temperature range, excellent tear and abrasion resistance, good resistance to non-polar solvents, high compression, tensile strength, and low-temperature elasticity. Therefore, TPUs are of great importance not only from an industrial but also from an academic point of view [3].

Similarly as of all polymeric materials, most of the TPUs properties are strongly dependent on temperature, which at different values induces different conformational changes in the polymer chains, leading to the corresponding transition processes that determine the behavior of polymers at various conditions [4]. In this sense, the influence of temperature on dynamic mechanical properties and visco-elastic behavior of TPUs plays a considerably important role and it is a longstanding subject of interest for many researchers. Nowadays, one of the most sensitive and most accurate methods used for study of dynamic mechanical properties, as well as of visco-elastic behavior of polymeric materials under various conditions (such as temperature, time, frequency of dynamic mechanical loading, and many others [5]), is represented by the dynamic mechanical analysis (DMA).

### 1.1. Dynamic Mechanical Analysis

**Figure 1:**
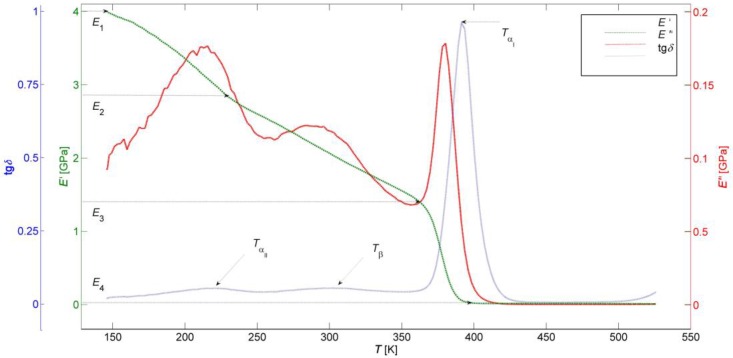
Temperature dependences of the average storage modulus *E*′(*T*), loss modulus *E′′*(*T*), and damping factor tan δ(*T*) of tested thermoplastic polyurethanes (TPU) at a frequency of 1 Hz in the whole temperature range of its service life.

DMA is a testing and analytical technique that is commonly used to characterize the mechanical responses of materials subjected to periodic dynamic stress in a cyclic manner, mostly the sinusoidal deformation force with very small amplitude. This technique helps engineers and scientists to understand and subsequently to predict the long-term material behavior under cyclic loading conditions. In the DMA test of polymeric material, a weak sinusoidal stress is applied at various frequencies and the strain inside a specimen of the known geometry is recorded as the temperature increases, which ultimately allows determination of the storage modulus *E′*, loss modulus *E′′*, and the damping factor tan δ, as well as the identification of relaxation events that occurred in the tested polymer sample following the transition processes due to the conformational changes of its macromolecular chains. The storage modulus (stiffness) provides a measure of elastic energy stored in the material, the loss modulus (energy absorption or damping) refers to the amount of energy dissipated in the form of heat in each cycle of the sinusoidal deformation, while the ratio of the loss modulus to the storage modulus gives the damping factor. Due to the visco-elastic nature of the polymeric material, the sinusoidal strain lags the applied stress by a phase angle δ, which is a measure of the visco-elastic damping or internal friction. The results obtained by investigations of dynamic mechanical properties or of visco-elastic behavior of polymeric materials using the DMA method are typically provided as a graphical representation of *E′*, *E′′* and tan δ versus temperature or time at one or more frequencies of dynamic mechanical loading (Figure 1). It is then possible to identify in those diagrams all of the relaxation transitions that occurred in the tested polymer at corresponding transition temperatures [6].

### 1.2. Relaxation Transitions in Polymers

A key relaxation process in polymers is a glass transition at a temperature, *T*_g_, which is frequently referred to as an α-transition, α-relaxation or as a primary relaxation. The *T*_g_ or *T*_α_ point is defined as a temperature, at which the amorphous portion of polymeric materials experiences a physical change from a hard and brittle glass-like condition to very flexible rubbery-like state. Due to the fact that the glass transition event covers a range of temperatures without discontinuity in the measured, *T*_g_, glass transition temperature is generally taken as a mid-point of this range. For amorphous portion of polymers, besides the glass transition, also one or more sub-*T*_g_ phenomenon often referred to as secondary relaxations, can be observed. They are referred to as β-, γ- and δ-transitions at the temperatures *T*_β_, *T*_γ_ and *T*_δ_ and they appear in order of the descending temperature. These transition processes are the result of local segmental motions that occur in a glassy state. By ‘local’, it is meant that only a small group of atoms are involved in the sub-*T*_g_ processes, the pure existence of which proves that material in a glassy state is a dynamic one. The glass transition occurs when large segments of polymer chains start moving, β-transition is related to either localized movement in the main chain or the large side movement, and γ-transition relates with the movements occurring in the side chain of polymers. The δ-transition is related to very small motions within macromolecule [7]. 

The authors Huh and Cooper in the work [8] have reported that TPUs may exhibit two separate glass transitions in the hard-and-soft-segment domains, respectively. The lower glass transition temperature *T*_αII_ (*T*_γ_) is associated with a change from stiff glassy behavior of the soft segments to their soft rubbery behavior and the higher one *T*_αI_ (*T*_α_) is due to the breakdown of hydrogen bonded interactions of Van der Waal’s forces between the rigid and flexible segments of TPUs. 

Above the glass transition temperature, upon reaching the flow temperature *T*_f_, the complete macromolecular chains can slip against each other. The amorphous structures of the polymer start to melt without chemical degradation of macromolecules. When the crystallite melting temperature *T*_m_ is reached, the crystallites also start to melt. Due to the fact that the temperature range of crystallite melting exceeds the flow temperature of the amorphous state, the entire polymeric material will be plasticized. As long as no thermal degradation will occur in the molten phase at the decomposition temperature *T*_d_, the polymer can reversibly get back into the solidified state by cooling and crystallite phases will again be generated. However, the size and distribution of crystallites can be now differing from the original status [9]. The crystalline and amorphous fraction of the semi-crystalline polymer below the *T*_g_ temperature are present in the glassy state, within the temperature range between *T*_g_ and *T*_m_ the amorphous fraction will be in the rubbery state and above the temperature *T*_m_ the crystalline fraction of the polymer will also already pass into the rubbery state, while the highly crystalline polymers do not have the rubbery area. The higher the amorphous fraction in the polymer, the stronger is its rubbery area. At a melting point of the crystalline phase, which is lower than the flow temperature of the amorphous phase *T*_f_ and only in exceptional cases it is identical, the polymer suddenly passes from the rubbery state to the plastic state. The interval between the temperatures *T*_m_ and *T*_f_ depends on the molecular mass, the degree of crystallinity, as well as on several other parameters. In the case of a high share of the crystalline phase the importance of *T*_g_ disappears because the polymer retains the properties of elastic body with the low deformation ability (glass) till the phase transition at *T*_m_ temperature. However, the glass transition, melting of crystallites and thermal degradation of macromolecular chains are typical phase transitions virtually of all semi-crystalline polymers, including the TPUs [10].

### 1.3. Stiffness-Temperature Model of Thermoplastic Urethanes

The authors Mahieux and Reifsnider in the work [11] showed, that the temperature dependence of the storage modulus *E′*(*T*) at a given constant frequency of dynamic mechanical loading can be quantitatively described with high level of reliability by physically well substantiated unified analytical model based on a Weibull distribution of the breakage of secondary bonds between macromolecular chains during the primary and secondary relaxation processes in any polymeric systems in the form of

(1) $$E^{\prime }\left(T\right)=\sum_{i=1}^{N}\Delta E_{i}^{\prime }e^{-{\left(\frac{T}{\Theta_{i}}\right)}^{m_{i}}},$$

where *E′*(*T*) is the temperature dependent storage modulus and *T* is the absolute temperature of polymer, Δ*E′_i_* are the storage modulus magnitudes of particular transition steps, the coefficients *Θ_i_* represent absolute transition temperatures, the parameters *m_i_* are the Weibull moduli corresponding to the statistics of the secondary bond breakage, and *N* is the number of relaxation events observed in the investigated polymeric material. This model has been transformed later into a robust physically based analytical model for prediction of the stiffness modulus for a wide range of temperatures and frequencies/strain rates by Richeton et al. in the study [12].

The stiffness-temperature model, presented by the Equation (1), in the form of exponential temperature function

(2) $$E^{\prime }\left(T\right)=\left(E_{1}^{\prime }-E_{2}^{\prime }\right)\;e^{-{\left(\frac{T}{T_{\gamma }}\right)}^{m_{\gamma }}}+\left(E_{2}^{\prime }-E_{3}^{\prime }\right)\;e^{-{\left(\frac{T}{T_{\beta }}\right)}^{m_{\beta }}}+\left(E_{3}^{\prime }-E_{4}^{\prime }\right)\;e^{-{\left(\frac{T}{T_{\alpha }}\right)}^{m_{\alpha }}}+E_{4}^{\prime }\;e^{-{\left(\frac{T}{T_{f}}\right)}^{m_{f}}},$$

where *E′*_1_ is the instantaneous storage modulus at the beginning of *E′*(*T*) DMA curve, *E′*_2_ is the instantaneous storage modulus immediately after the γ-transition, *E′*_3_ is the instantaneous storage modulus immediately after the β-transition and *E′*_4_ represents the instantaneous storage modulus at the beginning of the rubbery plateau (Figure 1), was successfully applied in one of our previous works in an analytical description of changes in the storage modulus of the tested TPU over a wide range of temperatures [13]. Both unknown physical parameters of the stiffness-temperature model (dynamic storage moduli and transition temperatures), as well as its statistical parameters (Weibull moduli) were estimated in the process of parametric fitting of the Equation (2) to the experimental *E′*(*T*) DMA curve using a trust region algorithm for a non-linear least squares method. However, critical values of the storage modulus *E′*_1_ − *E′*_4_ can be determined from the *E′*(*T*) DMA curve (Figure 1), the transition temperatures *T*_α_, *T*_β_, *T*_γ_ from the local maxima of the tan δ(*T*) curve (Figure 1) and the flow temperature *T*_f_ from the onset of the constant value of *E′* on the *E′*(*T*) curve (Figure 1). Accordingly, in the process of the multi-parametric fitting of experimental data set it is enough to estimate the unknown Weibull moduli *m*_α_, *m*_β_, *m*_γ_ a *m*_f_, which quantify the width of the secondary bonds failure distribution corresponding to the level of crystallinity (*m*_α_), the width of the secondary bond strengths distribution in the given temperature intervals (*m*_β_, *m*_γ_), and the width of secondary bond strength distribution corresponding to the TPU degree of cross-linking (*m*_f_) [14]. At the same time, the temperature *T*_αII_ (*T*_γ_) is associated with the glass transition of TPU soft segments, *T*_αI_ (*T*_α_) with the glass transition of its hard segments, *T*_β_ can be attributed to a short-range order translation and to re-orientational motions within the PTU crystals, whereas *T*_f_ corresponds to global translations of whole polymer molecules between entanglements [15]. In our work [13], it has been shown that the stiffness-temperature model presented by the Equation (2) is very successful in describing variations of the elastic behavior of the tested TPU with temperature from its glassy state up to the flow region and within the property transition regions. At the same time, this model makes it possible to predict the stiffness-temperature dependence of different types of TPUs, including the newly proposed ones, without necessarily having to subject them to time demanding and costly DMA testing.

Unfortunately, there is currently no physically justified unified analytical model that would be able to reliably describe also the viscous behavior of polymeric systems in the whole temperature range throughout their service life, including all of the observed relaxation transitions in a way that Equation (1) describes their elastic behavior. However, the authors Ying Li et al. in work [16] present a predictive multi-scale computational framework to evaluate the visco-elastic properties of linear polymers based on their atomistic constituents. Many different multi-scale computational techniques are described in detail in [17]. Whereas, the artificial neural networks (ANNs) are, in general, excellent universal approximators not only of linear but also of non-linear functional dependencies between dependent and independent variables [18], it is quite natural to assume that temperature dependence of the loss modulus *E′′*(*T*) and damping factor tan δ(*T*), as well as that of the storage modulus *E′*(*T*), could be successfully modeled right by them. 

### 1.4. Artificial Neural Networks

ANNs are intelligent mathematical computational methods inspired by biological neural networks and human brain that can learn from experience and generalize the learned things. Today they are used especially in the cases, where classic techniques fail, as well as other solutions for such problems, which can be formulated in the form of associative tasks [19]. ANNs are especially efficient in approximation of any continuous function with a finite number of discontinuities by mapping the relationships between vectors of the input (independent) and output (dependent) data [20]. With the right choice of structure and architecture they are capable to learn on the basis of the well-chosen input-output patterns to also model very complicated non-linear relations between the input and output data, for example to model a highly non-linear mechanical behavior of the materials such as polymers and their composites [21,22]. However, the acquired knowledge is, unfortunately, hidden in such ANN structures that cannot readily be extracted and interpreted. ANNs with multi-layer feed-forward architecture are the most commonly used neural networks in engineering applications thanks to their structural simplicity and relatively fast learning abilities [23].

The multi-layer feed-forward ANNs are non-parametric and robust mathematical models that are designed to find non-linear relationships between specified input-output data samples. This type of ANNs consists of at least of three layers of artificial neurons or processing units. The first layer is the input layer and the last layer is the output layer, while all of the layers between the input and output layers are referred to as the hidden layers. In this architecture of the ANNs, each neuron is connected to all of the neurons in the next layer but there are no connections between the neurons on the same layer. The signal propagates from the input layer, through all of the hidden layers, to the output layer in a forward direction. The numbers of neurons in the input, as well as in the output layers, are determined by the formulation of the investigated problem. The number of hidden layers and the number of neurons in these layers are related to the complexity of the relevant information contained in the learning or training data set. At the same time, the number of neurons in the hidden layers defines the capacity of the ANN. The knowledge about the relations between the input and output data is stored in the weights of connection between neurons, the values of which are updated during the supervised training process of the ANN with a set of known and representative values of the input-output data samples [24]. The training process starts with randomly set weights that are gradually updated by the minimization algorithm of squared difference between the target value and the computed output value on the output of each ANN neuron. Generally, to update the network weights iteratively, a technique called the back-propagation algorithm is used, which involves performing computations backward through the ANN [25]. The aim of the research described in the present work is exactly the modeling of the temperature dependencies of *E′*(*T*), *E′′*(*T*), and tan δ(*T*) of the investigated TPU submitted to DMA tests at a constant single frequency of dynamic mechanical loading in the whole temperature interval of its service life, or from its glassy state up to the flow region, using the multi-layer feed-forward ANNs utilizing the back-propagation learning algorithm.

## 2. Materials and Methods

Most of conventional TPUs are based either on polyester or polyether polyols, 4,4-diphenylmethane diisocyanate as an isocyanate component, and 1,4-butanediol as a chain extender [26]. In the present work, the synthesis of the tested TPU was performed in one liter of glass reactor by mixing two commercial reactive components—Axson Technologies PX 522/HT POLYOL and Axson Technologies PX 521-522 HT ISO ISOCYANATE (Axson Technologies, Newmarket Suffolk, UK)—under normal pressure and at room temperature. In order to ensure degassing and homogenization before polymerization, the reactive mixture was mixed for approx. 5 min with the use of an ultrasound mixer WELDER (WELDER, Hiroshima, Japan) in a VB vacuum chamber (VB, Prague, Czech Republic). Subsequently, the mixture was poured into a mold and left to cure at 80 °C for 4 h. The post-curing process proceeded at 100 °C for 16 h. Under these conditions the addition of catalyst was not necessary. From the resulting material, ten rectangular shaped samples with dimensions of 30 mm (length) × 6 mm (width) × 1.5 mm (thickness) were cut off using a Gravograph LS100 40 W CO_2_ laser cutter (Gravograph, Paris, France). The samples were then subjected to dynamic mechanical analysis over the entire temperature range of the TPU service life at a single constant frequency of dynamic mechanical loading. 

The DMA test measurements were carried out using a Perkin Elmer Pyris Diamond 8000 Dynamic Mechanical Analyzer (Perkin Elmer, Baesweiler, Germany) in the uniaxial tensile mode at a constant frequency of dynamic mechanical loading of 1 Hz, with an amplitude of 20 μm from −125 up to 250 °C and at a constant heating rate of 3 °C·min^−1^. The amplitude of applied dynamic stress was 0.1 MPa and the strain rate was 0.1 s^–1^. The strain measurements were performed by a linear variable differential transformer in the DMA equipment with a span of 2 mm and a mean resolution of 2 nm. The measurements were performed on ten specimens cooled by liquid nitrogen from a Dewar flask (Perkin Elmer, Baesweiler, Germany). The average values of *E′*, *E′′*, and tan δ for each temperature *T* were computed. The measurement uncertainty was approx. 2%. The experimental results of DMA tests of the examined material in the form of temperature dependencies of the average storage modulus *E′*(*T*), loss modulus *E′′*(*T*), and damping factor tan δ(*T*) at a single constant frequency of dynamic mechanical loading of 1 Hz in the whole temperature range of its service life are presented in Figure 1. The frequency of 1 Hz was chosen due to the fact that the secondary transitions and other structural features of the tested material could be easily detected, and the obtained results could be in the case of need more easily related to the experimental data acquired with the use of other techniques (e.g., by DSC method [27]). Figure 1 shows also highlighted critical values of the storage modulus *E′*_1_ – *E′*_4_ and temperatures of relaxation events *T*_αII_, *T*_β_, *T*_αI_ occurred in the tested TPU samples. In connection with the above described stiffness-temperature analysis of experimental data, the temperatures in the diagram in Figure 1 are presented in the absolute scale. 

### Artificial Neural Network Modeling

This work aimed at an investigation of temperature dependencies of the observed visco-elastic parameters *E′*(*T*), *E′′*(*T*), and of tan δ(*T*) of the investigated TPU at a given constant frequency of its dynamic mechanical loading, three multi-layer feed-forward artificial neural network models were created utilizing the error back-propagation learning algorithm with an architecture consisting of one input, one hidden and one output layer [28]. The temperature history *T*(*t*) of TPU during the DMA test, representing a data set of the input experimental data, was naturally chosen as the sole input parameter of the ANN for all three of the created models, while the corresponding experimental values of the storage modulus, loss modulus, as well as damping factor, were chosen as a target ANN data set for each model individually. The input and output layer has one neuron, which corresponds with the number of the input and output variables of individual models, while it was necessary to optimize the number of neurons in a hidden layer with the use of a trial and error method for each individual model separately. The main criteria for this optimization were the maximum network performance and its trouble-free learning. A single hidden layer was chosen in view of the practical requirement for the maximum network simplicity, its high stability, trouble-free learning, and minimal demands on computing power and computing time. Moreover, a number of mathematical works has shown that virtually any of the above described functions can be approximated with the required precision by means of a well-trained feed-forward ANN with a single hidden layer under the mild assumptions of the activation function (transfer function), which follows from the so called universal approximation theorem for feed-forward ANNs [29].

Between the input and hidden layer of the ANN a hyperbolic tangent sigmoid transfer function was used in the form of [30]

(3) $$\tan sig\left(n\right)\;=\;\frac{1-e^{-2n}}{1+e^{-2n}},$$

where *n* represents the net input into the hidden layer transfer function, which keeps the neurons output between certain limits as it is the case in the biological neuron. In order to avoid limitation of the network output by a small range of values, the pure linear transfer function

(4) purelin(m)=m,

where *m* is the net input into the transfer function of the output layer, was used between the hidden and output layer [30].

Generally, each neuron of the ANN represents its elementary building and computing unit located in the appropriate network layer. The input layer only transmits the input signal **p** to the hidden layer neurons without performing any calculations. Neurons of the hidden and output layer contain input, bias, weight, summing junction, transfer function, and output. At the input of each neuron of the hidden layer, each element *p_i_* of the input data vector **p** with the length *R*^1^ is multiplied by its weight *iw*^1,1^. The weighted inputs of all *S*^1^ neurons of the hidden layer are summed in their summing junctions. This weighted sum of the inputs together with the added bias *b*^1^*_j_* of the current neuron create an activation potential

(5) $$n_{j}^{1}=\sum_{i,j}iw^{1,1}p_{i}+b_{j}^{1}$$

leading to the activation of its output, while the bias is just a constant external input with a value of 1 multiplied by its weight, which represents the basic level of activity of the given neuron [31]. The activation signal, given by the Equation (5), or the net input of the hidden neuron *n*^1^*_j_* is then fed as the only argument into the non-linear transfer function, given by the Equation (3), which generates its output

(6) $$a_{j}^{1}=f^{1}\left(n_{j}^{1}\right)=\tan sig\left(n_{j}^{1}\right)=\frac{1-e^{-2n_{j}^{1}}}{1+e^{-2n_{j}^{1}}}\;.$$

The total output of the hidden layer with *S*^1^ hidden neurons, represented in a matrix form as

(7) $$a^{1}=f^{1}\left(IW^{1,1}p+b^{1}\right),$$

is then sent to the output layer. 

Net input of the output layer

(8) $$n_{j}^{2}=\sum_{j}lw^{2,1}a_{j}^{1}+b_{j}^{2},$$

created by summing in the summing junction, is then inserted into the pure linear transfer function given by the Equation (4), which returns the output signal

(9) $$a_{j}^{2}=f^{2}\left(n_{j}^{2}\right)=purelin\left(n_{j}^{2}\right)=n_{j}^{2},$$

or the output signal represented in a matrix form

(10) $$a^{2}=f^{2}\left(LW^{2,1}a^{1}+b^{2}\right).$$

Total output signal of the network **y** can be then presented as a matrix in the form

(11) $$y=a^{2}=f^{2}\left(LW^{2,1}f^{1}\left(IW^{1,1}p+b^{1}\right)+b^{2}\right).$$

The weight matrix **IW^1,1^** represents a matrix of weights between neurons of the hidden and input layer, while **LW^2,1^** represents the weight matrix between neurons of the hidden and output network layers. The upper index in the designation of all of the network elements represents its respective layer, it means that index ^1^ denotes a hidden layer and index ^2^ denotes an output layer. The lower index *_i_* belongs to the elements of the input vector **p**, while the lower index *_j_* corresponds to the individual neurons in the respective layer (indicated by the upper index).

Due to the fact that the output of the hidden layer **a^1^** is also the input of the output layer with the output **a^2^**, the hidden layer can be, in general, analyzed also as a single layer network with *S*^1^ inputs, *S*^2^ neurons and *S*^2^ × *S*^1^ weight matrix

(12) $$W^{2}=\left|\begin{array}{ll} w_{1,\;1}^{2} & w_{1,\;2}^{2}\;\cdots \;w_{1,\;S^{1}}^{2}\\ w_{2,\;1}^{2} & w_{2,\;2}^{2}\;\cdots \;w_{2,\;S^{1}}^{2}\\ w_{S^{2},\;1}^{2} & w_{S^{2},\;2}^{2}\;\cdots \;w_{S^{2},\;S^{1}}^{2} \end{array}\right|.$$

with weights *w*^2^_*r,q*_, where the lower index *_q_* denotes an element of the input vector **a^1^**, while the lower index *_r_* denotes the appropriate neuron, into which the signal from this element is brought [32]. Each neuron of the hidden layer of the proposed ANN is connected by means of weights with each element of the input vector and with neuron of the output layer, and by bias also with external environment of the network.

**Figure 2:**
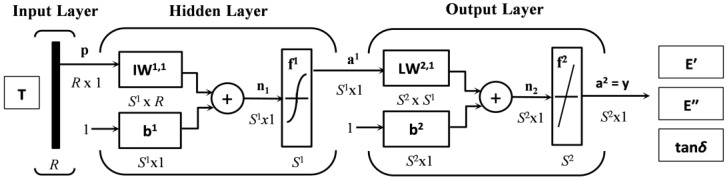
Schematic of the artificial neural networks (ANN) architecture for modeling the temperature dependencies of the storage modulus *E′*(*T*), loss modulus *E′′*(*T*) and damping factor tan δ(*T*) of the investigated TPU at a given constant frequency of its dynamic mechanical loading.

The ANN architecture for modeling the temperature dependencies of all three observed visco-elastic parameters is in an abbreviated notation [32], schematically shown in Figure 2. The input vector **p** in our case for all three generated ANN models represents a vector of the temperature history **T** of the TPU during its DMA test, while the output signal **y** of the network for individual models are the vectors of the corresponding values *E′*(*T*), *E′′*(*T*), or tan δ(*T*) at temperatures, *T*(*t*) predicted by the network. Vectors and matrices are in this work marked in straight bold fonts.

At first, it was necessary to teach the created ANN to recognize the relationships between the input and output data of the DMA test, then to verify the reliability of the learned recognition and finally to test its ability to generalize the acquired and verified knowledge at prediction of new experimental results of the DMA test. The input and target data of the created ANN were therefore randomly divided to training, validation, and testing data sets in the proportion 0.70:0.15:0.15, respectively. There is no principle limitation for these divisions, but in generally, it is common that the training data set has larger amounts of data than the other sets [33]. On the other hand, the number of training data can strongly influence the ANN predictive quality [34].

The training data were used for supervised learning of the network [35], during which the difference between the values *y_k_* of the output vector **y** predicted by the network and the values *d_k_* of the vector of the desired (training) data **d** is minimized with the use of step by step modification of the weights **IW^1,1^**, **LW^2,1^** and biases **b^1^**, **b^2^**. Values of the error vector

(13) δ=d−y

is appreciated with the use of the mean squared error (MSE) function

(14) $$E\left(w,\;b\right)=\frac{1}{2l}\sum_{k=1}^{l}{\left(d_{k}-y_{k}\right)}^{2}\;,$$

where *l* is the total number of its elements, which is minimized during the ANN training process [36], which leads to an adaptation of weights and biases of the network to the training data, or to its optimization of the training data set.

The Levenberg–Marquardt back-propagation minimization algorithm [37], also known as the damped least squares method, based on the modification of gradient Gauss–Newton method [38], was used for training of all three ANN models. The iterative scheme of the Levenberg–Marquardt algorithm for the modification of weights and biases with the error function MSE has the form:

(15) $$z_{k+1}-z_{k}={\left(J_{k}^{T}J_{k}+\lambda_{k}I\right)}^{-1}J_{k}^{T}E_{k},$$

where *z* represents the weights *w* and biases *b* of the network, the product **J^T^***_k_* **J***_k_* is referred to as Hessian matrix approximation for non-linear least square optimization [39], **J***_k_* is the Jacobian matrix that contains first derivatives of the network MSE with respect to the weights and biases [40], **I** is the identity matrix [41], *λ_k_* is the non-negative learning parameter [42] and the symbol **^T^** {\displaystyle ^{\mathsf {T}}}denotes the matrix transpose [43]. The iterative learning process starts with a random adjustment of the weight and its adaptation continues until reaching the specified maximum number of iterations (epochs), or until the MSE network is minimized to a pre-determined target value. The learning process, during which the ANN seeks through the step-by-step modification of weights and biases the most accurate training data model, must be terminated before the network also begins to adapt to the noise present in each experimental data, which causes a dramatic reduction of its predictive abilities for the unknown data. Therefore, during minimization of the error function, a development of the error function in the validation data set is also monitored. In the event that error of the validation data begins to increase during several iterative cycles, while the error of the training data is still decreasing or is no longer changing, the training process is prematurely terminated in order to prevent the so called overfitting of the network, the cause of which is usually too high a number of neurons in the hidden layer [44]. The weights and biases, at which the MSE of the validation data set was minimal, are then taken as the resulting values. It means that the training data set is used to fit the model by finding its unknown but correct weights and biases, whereas the validation data set is used to test how well the model performs on the unseen data, or for debugging the ANN architecture (the number of hidden neurons or possibly also hidden layers).

The testing dataset contains the data, with which the ANN did not come into contact during the learning process or during validation, and which serve for verification of the level of network learning or for verification of its ability to generalize the acquired knowledge stored in the values of weights and biases values at prediction of new experimental results of the DMA test at new temperatures, unknown to the network. A significant difference in the number of iterations needed to achieve a minimum MSE of validation and testing data may indicate an inappropriate ratio of the division of the input-output data of the network to the training, validation, and testing data. The inability of the network to describe more significant non-linearities in the testing data set, or the so called ANN underfitting, is usually caused by an insufficient number of hidden neurons or hidden layers [44].

The ANN adaptation begins with random values of weights, that is why it is quite natural that each individual solution found in the process of its optimization may be completely different from any other, and in general, it is not possible to guarantee that the solution found is the best possible one. Even very different setting of the ANN weights can provide a practically identical prediction for training data, but the difference may occur in quality of the prediction for the unknown data. It is therefore always reasonable to repeat the training, validation, and testing of the ANN several times and to choose the model with the smallest MSE of the training, validation, and testing data.

The only reliable way to find an optimal number of neurons in the hidden ANN layer is the trial and error method. In this work, the initial values *N*_h_, calculated according to the empirical relation below, were used for an effective search of an optimal number of hidden neurons

(16) $$N_{h}=\frac{N_{s}}{\alpha \left(N_{i}+N_{o}\right)},$$

where *N*_s_ is number of the samples in the training data set, *N*_i_ number of the input neurons, *N*_o_ number of the output neurons, and α is a scaling factor with a value between 2–10, expressing the requirement of how general the ANN model should be, or to what extent the possibility of the network overfitting must be suppressed. In this work, the network training, validation and testing process for each selected *N*_h_ was repeated 50 times. As the optimal number of neurons in the hidden layer was taken the one, at which the MSE for all three data selections was minimal and the value of the linear correlation coefficient *R* between the output and the desired data

(17) $$R=\frac{N_{s}}{\alpha \left(N_{i}+N_{o}\right)},$$

approached the value of one in the closest possible manner. The covariance of the output and desired data is marked by the symbol *Cov*(*d*,*y*), while the symbols *s_d_* and *s_y_* represent their standard deviations, respectively [45].

The design of the structure and optimization of the ANN architecture for modeling of temperature dependencies of the storage modulus, loss modulus, and damping factor of the investigated TPU were performed in the development environment “Neural Network Toolbox“ of the software package Matlab^®^ Version 7.10.0.499 R2010a 64-bit (MathWorks, Natick, MA, USA), which provided a complete set of tools for sufficiently efficient work with artificial neuron networks of various types [32]. As it was already mentioned above, the Levenberg-Marquardt algorithm was used for training the network, the input-target data of which were randomly divided to training, validation and testing data sets according to the ratio given above. The various parameter settings for the ANN training are the maximum number of epochs to train: 1000, minimum performance gradient: 1^−10^, initial value for learning parameter: 1^10^, and the maximum validation failures: 6.

## 3. Results

Figure 1 shows the results obtained from DMA tests of the studied material. The *E′*(*T*), *E′′*(*T*) and tan δ(*T*) curves exhibit a typical shape for the high molecular weight, weakly semi-crystalline, thermoplastic elastomers throughout the temperature range below the flow region. The α-, β- and γ-transitions occur as a direct result of the three phase morphology, in particular crystalline hard segments phase, crystalline soft segments phase, and amorphous soft segments phase of TPU. The temperature *T*_αI_ corresponds to the glass transition of soft segments, *T*_αII_ corresponds to the glass transition of hard segments, and *T*_β_ corresponds to the short-range order translation and reorientational motions within the crystals [8]. A sharp drop of *E′*(*T*) at temperatures higher than *T*_αII_ indicates a significant phase separation induced by the symmetrical structure of rigid segments, a low crosslinking level and a small degree of crystallinity of TPU. The slope of the glass transition of the rigid segments, observed on the curve tan δ(*T*), shows the homogeneous structure of the physical network and suggests a relatively narrow variance of the molecular weights.

The temperature independent rubber plateau corresponds to uniform rigid segments forming stable crystals, which soften in a relatively narrow temperature range, as evidenced by the increase in tan δ(*T*) at the end of the DMA curve. It was proved in [13], that the stiffness-temperature model (2) is able to describe only the functional dependence *E′*(*T*) with a high degree of reliability, i.e., the elastic behavior of the examined TPU.

The ANN, the schematic of architecture of which is presented in Figure 2, provides for *E′*(*T*) the results with the lowest MSE and the highest accuracy *R* for all three data sets (training, validation and testing) simultaneously at 14 neurons, for *E′′*(*T*) at 13 neurons and for tan δ(*T*) at 15 neurons in a hidden layer. Taking into account that the input and output layer of all three ANN models contains a single neuron, the optimal architecture of the multi-layer feed-forward back-propagation ANN for modeling of temperature dependence of the storage modulus *E′*(*T*), loss modulus *E′′*(*T*) and damping factor tan δ(*T*) of the investigated TPU at given constant frequency of its dynamic mechanical loading in the whole temperature range of its service life can be presented in numeric form as 1-14-1, 1-13-1 and 1-15-1, respectively [46].

**Figure 3:**
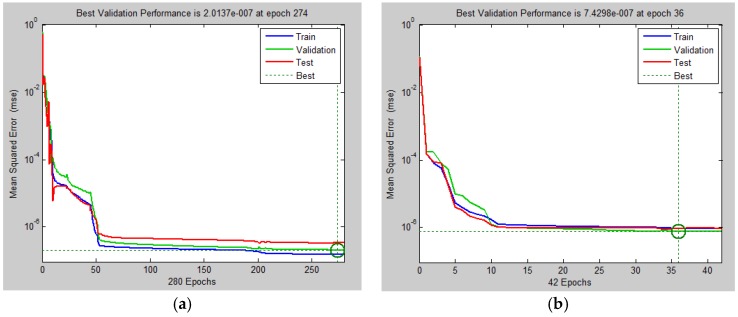

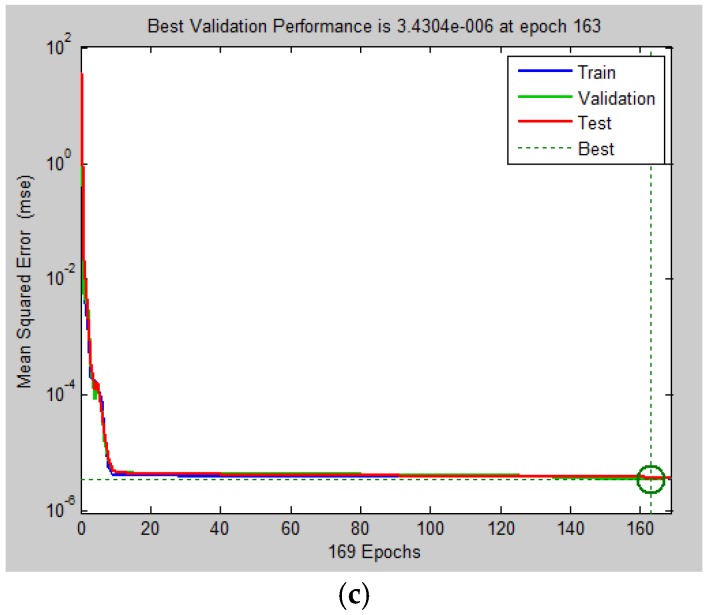
(**a**) Performance plots of the ANN models for *E′*(*T*); (**b**) Performance plots of the ANN models for *E′′*(*T*); and, (**c**) Performance plots of the ANN models for tan δ(*T*).

The ANNs do not usually enable a calculation of statistical parameters and diagnostics for a detailed assessment of the model quality, with the use of for example variance of regression coefficients, F and t statistics for the testing the significance of the model, diagnostic graphs, etc., as it is in the case of parametric regression models of experimental data [47]. For this reason, it is necessary to use different methods for the assessment of quality of the ANN model [48]. In this work, in order to validate the ability and accuracy of the modeled results related to all of three investigated visco-elastic parameters, first of all of the performance plots of the ANN models have been constructed and they are shown in Figure 3. These plots show how the MSE function minimizes during the training process. After six validation checks the network stops its training and returns to the state, in which the minimum validation performance was observed. This is indicated by the green circle in each figure. It follows from the plot in Figure 3a that the best validation performance of the ANN model for *E′*(*T*) occurred at the epoch 163 with the associated MSE value of 3.4304 × 10^−6^, for *E′′*(*T*) at the epoch 36 with MSE value of 7.4298 × 10^−7^ (Figure 3b) and for tan δ(*T*) at the epoch 274 with MSE value of 2.0137 × 10^−7^ (Figure 3c). The final MSE of all three ANN models is rather negligible, the training data set MSE, the validation data set MSE, as well as the test data set MSE have similar characteristics. No significant overfitting has occurred by iteration (where the best validation performance has occurred), which has indicated the achievement of excellent results for both the approximation and the prediction of values of the investigated functions [49]. However, the training of the ANN model for *E′′*(*T*) is the least time-consuming, as this model converges to the minimal MSE value already in the epoch 36 of the training process, while the most time-consuming training of the ANN model for tan δ(*T*) converges to the MSE minimum only in the epoch 274.

**Figure 4:**
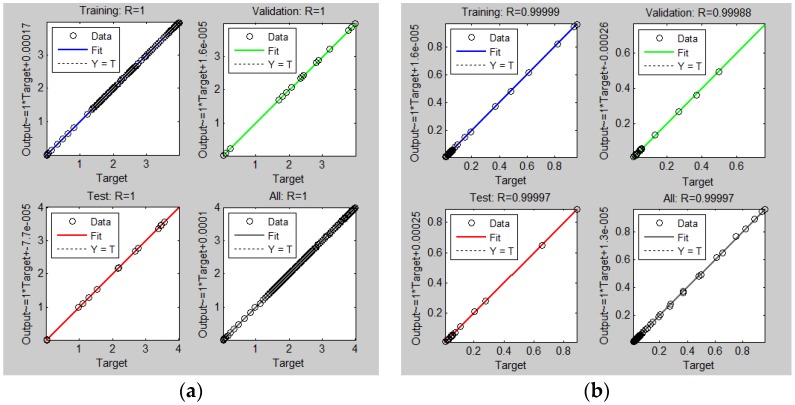

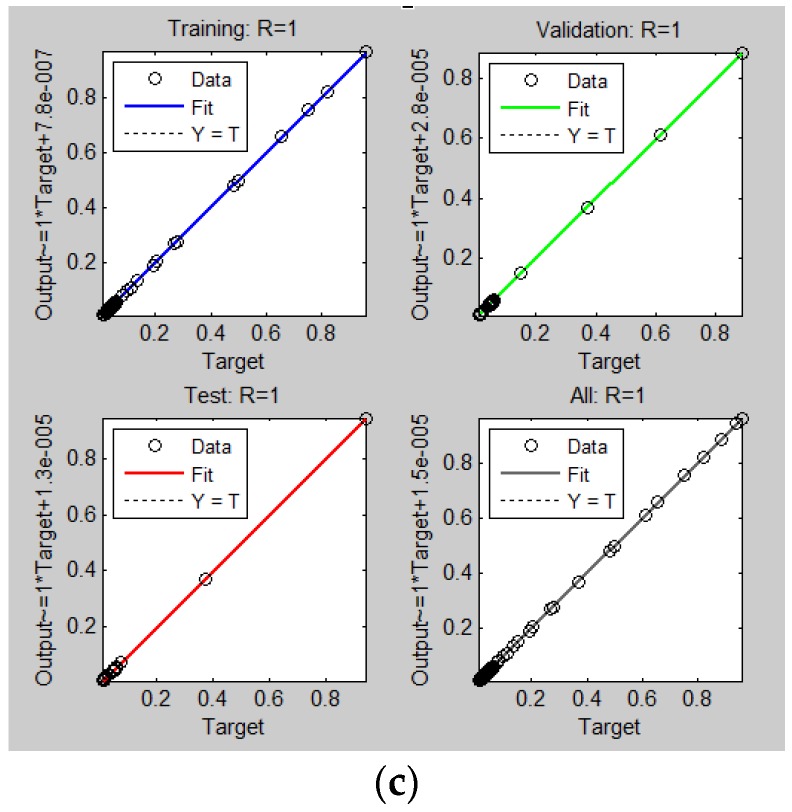
(**a**) Fitness plots for training, validation, testing and all data sets of the ANN to predict *E′*(*T*); (**b**) Fitness plots for training, validation, testing and all data sets of the ANN to predict *E′′*(*T*); and, (**c**) Fitness plots for training, validation, testing and all data sets of the ANN to predict tan δ(*T*).

The MSE function is a performance measurement adopted to determine the ANN performance, while *R* values measure the correlation between the outputs and targets. In Figure 4, the linear regression plots of targets relative to outputs or fitness plots for all three data sets of the ANN (training, validation, and testing) and entire data used in the network models for *E′*(*T*) (Figure 4a), *E′′*(*T*) (Figure 4b), and tan δ(*T*) (Figure 4c) are presented. In the top and left part of these figures the *R* values and equations of linear fit lines in the format

(18) Output~=slope⋅Target+intercept

are shown, respectively.

It is apparent from the linear regression plots presented in Figure 4, which practically all of the modeled data (hollow circles) fall on a 45° line expressing the exact agreement between the computed values (output data) and experimental ones (target data) with the associated *R* value of 1, which confirms the achievement of excellent results of all three analyzed ANN models [44]. Lower values of the correlation coefficients *R* in the case of *E′′*(*T*) of the ANN model in comparison with other two models document, that the nonlinear behavior of TPU damping under the given conditions is higher than that of its elastic resistance or stiffness. Namely, the different level of non-linearity of the temperature dependencies of individual monitored visco-elastic parameters is the reason why we did not propose for solution of our problem a single ANN model with three outputs but a single-output ANN model for each individual parameter separately.

**Figure 5:**
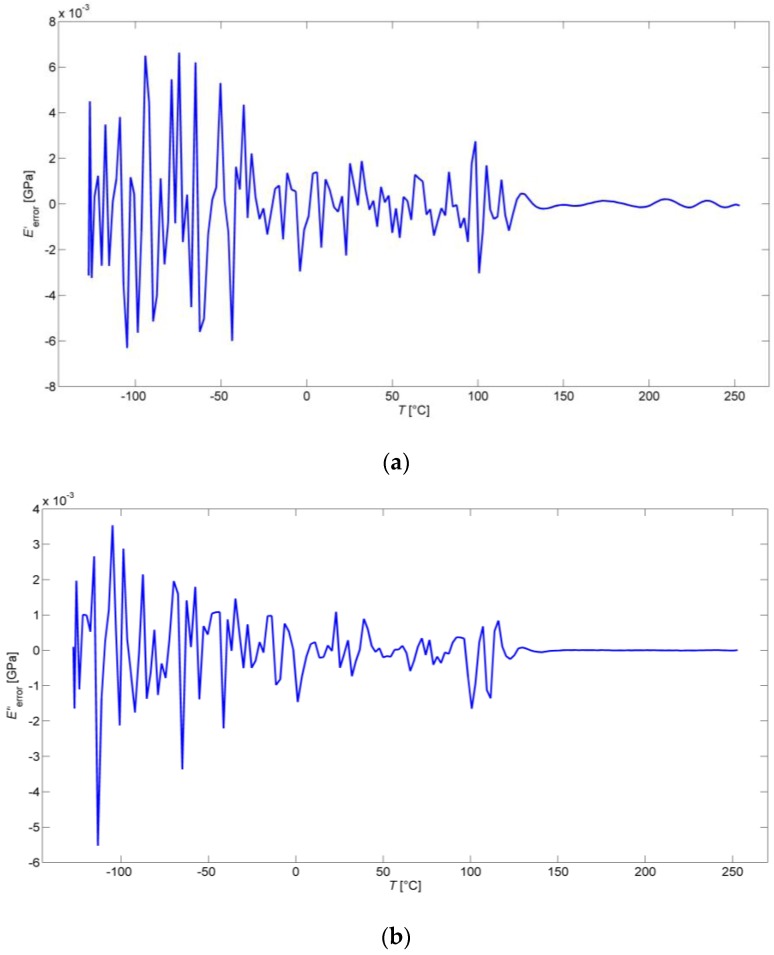

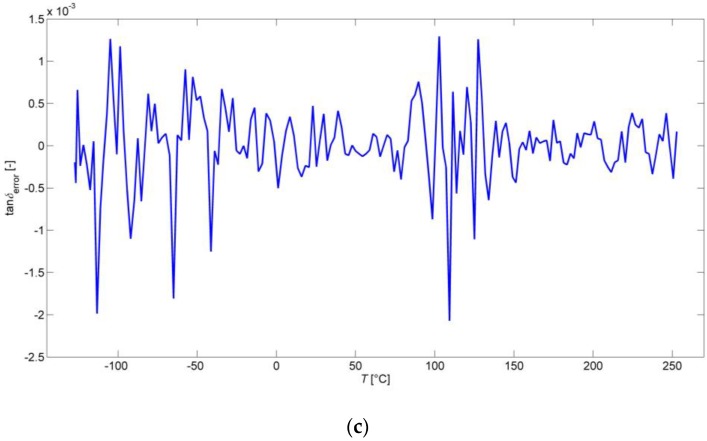
(**a**) Errors of the ANN model for *E′*(*T*); (**b**) Errors of the ANN model for *E′′*(*T*); and, (**c**) Errors of the ANN model for tan δ(*T*).

The difference between the experimental and modeled values of *E′*(*T*), *E′′*(*T*), and tan δ(*T*) are presented in Figure 5, in detail. It can be seen that error of all three ANN models on the level of the order of 10^−3^ is negligibly small in comparison with the experimental data acquired from the DMA tests.

After being sure about the accuracy and authenticity of the results, for any unknown value of the temperature from the whole investigated temperature range, the corresponding value of the storage modulus, loss modulus, as well as damping factor of the examined TPU can be obtained by simulation of the above described ANN models using the found numerical amounts of weights and biases for each layer [50].

## 4. Conclusions

In the presented work, the artificial neural network technique has been used to model the temperature dependence of dynamic mechanical properties and viscoelastic behavior of thermoplastic polyurethane over the wide range of temperatures. The variations of the storage modulus, loss modulus and damping factor with temperature, obtained from the dynamic mechanical analysis tests across transition temperatures at constant single frequency of its dynamic mechanical loading, have been modeled by three models of well-trained, multi-layer feed-forward back-propagation artificial neural network, utilizing the Levenberg–Marquardt learning algorithm, with one hidden layer, the hyperbolic tangent sigmoid transfer function between the input and hidden layers, and the pure linear function between the hidden and output layers. The excellent agreement between the modeled and experimental data has been found over the entire investigated temperature interval, including all of the observed relaxation transitions. The multi-layer feed-forward back-propagation artificial neural network has been confirmed to be a very effective artificial intelligence tool for the modeling of dynamic mechanical properties and for prediction of viscoelastic behavior of the tested thermoplastic polyurethane within the whole temperature range of its service life. The ANN is used by many researchers for the analysis of viscoelastic properties of polymers. There is no work up to date dealing with ANN modeling of dynamic mechanical properties and the viscoelastic behavior of TPUs, which are currently among the most used construction materials in many practical applications. The excellent predictive abilities of ANN models can also be used in the development of new TPUs-based materials and polymer composites with desired dynamic mechanical properties, relaxation transitions, and predictable viscoelastic behaviors.
